# Coupling Biocompatible Au Nanoclusters and Cellulose Nanofibrils to Prepare the Antibacterial Nanocomposite Films

**DOI:** 10.3389/fbioe.2020.00986

**Published:** 2020-08-18

**Authors:** Peng Wang, Baishuang Yin, Huiling Dong, Yibo Zhang, Yangheng Zhang, Rixin Chen, Zukun Yang, Caoxing Huang, Qing Jiang

**Affiliations:** ^1^State Key Laboratory of Pharmaceutical Biotechnology, Department of Sports Medicine and Adult Reconstructive Surgery, Nanjing Drum Tower Hospital, The Affiliated Hospital of Nanjing University Medical School, Nanjing, China; ^2^College of Chemical Engineering, Nanjing Forestry University, Nanjing, China; ^3^College of Animal Science and Technology, Jilin Agricultural Science and Technology University, Jilin, China; ^4^Jiangsu Engineering Research Center for 3D Bioprinting, Jiangsu, China; ^5^Department of Periodontology, Nanjing Stomatological Hospital, Medical School of Nanjing University, Nanjing, China; ^6^Jiangsu Key Laboratory of Oral Diseases, Nanjing Medical University, Nanjing, China

**Keywords:** cellulose nanofibrils, Au nanoclusters, antimicrobial property, wound-healing dressing, multipurpose films

## Abstract

Cellulose nanofibrils (CNF) is considered as an inexhaustible precursor to produce antibacterial materials, such as antibacterial hydrogel, antibacterial paper, and antibacterial film. However, the poor antimicrobial property of neat CNF required it should be coupled with an antibacterial ingredient. Herein, biocompatible Au nanoclusters (AuNCs) were synthesized and added into the CNF dispersion to prepare a novel antibacterial film (AuNCs@CNF film). The effects of addition of AuNCs with different amount on the morphology and physicochemical properties of AuNCs@CNF films were characterized using atomic force microscopy (AFM), scanning electron microscopy (SEM), X-ray diffraction (XRD), FTIR (Fourier-transform infrared), light transmittance spectra, and thermogravimetric analysis (TGA). The results showed that AuNCs did not affect the nano-structural features of the CNF film and its basic structures, but could greatly increase the hydrophilicity, the flexibility and the thermal stability of CNF film, which might improve its application in antimicrobial wound-healing dressing. The prepared AuNCs@CNF films demonstrated high antibacterial properties toward *Escherichia coli* (*E. coli*) and *Streptococcus mutans* (*S. mutans*) both *in vitro* and *in vivo*, which can prohibit their growths and promote the healing of bacteria-infected wound, respectively. Thus, the prepared AuNCs@CNF film with great antibacterial properties could be applicable in biomedical field.

## Introduction

Cellulose is the most abundant polysaccharide on earth and has been considered as an inexhaustible source to produce the environmental-friendly and biocompatible materials (Bian et al., 2019; Wu et al., 2019a; Lin et al., 2020; Liu et al., 2020). Recently, procuring cellulose nanofibrils (CNF) from cellulose has gained attention to produce nanofiber-reinforced composites, including microelectronic and electro-optical films, gas-barrier films, cosmetics, and food packing films (Rojo et al., 2015; Li et al., 2017; Zhang et al., 2019). The desired CNF possesses superior properties than the conventional cellulose derivatives, such as high specific surface area, ease of processing, good mechanical properties, biodegradability, and high transparency (Du et al., 2019; Bian et al., 2020). Thus, CNF is considered as the precursor to produce antimicrobial materials, such as antibacterial hydrogel, antibacterial paper, and antibacterial film, which can be used as the food packing films, drug carriers, infected wound-healing formulation and multifunctional antibacterial films (Li et al., 2018; Han et al., 2019). Since the neat CNF lacks the antimicrobial properties, it has limited application in biomedical fields. Hence, various technologies have been applied to improve the antibacterial properties of CNF-based materials, such as surface modification, antibiotic addition, combination with nanomaterials, and combination with antibacterial polymers (Jia et al., 2012; Li et al., 2018; Bagde and Nadanathangam, 2019).

Among the technologies for constructing the antibacterial CNF materials, adding antibiotics in CNF dispersion is the facile method to obtain the antibacterial CNF film for preparing the wound dressing materials (Kaplan et al., 2014). The CNF-based dressings have nanofiber structures with high capability to absorb and retain water, which enhances its ability to absorb wound exudate and control the environment for wound healing (Li et al., 2018). On the other hand, the used antibiotics can inevitably cause the emergence of multidrug-resistant bacterial strains, which is currently a major public health concern globally (Wright, 2011). To overcome this limitation, the biosecurity nanostructures, including metal, metal oxides, and carbon-based nanomaterials, are considered as promising platforms for antibacterial applications, such as infected wound-healing formulation and multifunctional antibacterial films (Han and Wang, 2017; Fardioui et al., 2018; Wahid et al., 2019). The antibacterial property of the engineered nanomaterials facilitates their usage as potential antibacterial alternatives to overcome the superbug and drug resistance infections (Zou et al., 2016; Fang et al., 2018). Elemental nanoparticles (NPs), such as Ag or Cu NPs with intrinsic antimicrobial capacity, have been extensively utilized to produce of antibacterial films (Chen et al., 2019). These NPs can react with the cell membrane protein by thiol groups, then induce the production of intracellular reactive oxygen species (ROS) or affect bacterial transport of substances through the cell membrane (Percival et al., 2005; Dizaj et al., 2014; Yuan et al., 2018). However, the inherent cytotoxicity for human cells restricts their biomedical application (Holt and Bard, 2005; El Badawy et al., 2011). Therefore, finding alternate nanoscale antibacterial candidates with good biosecurity for potentially clinical application is rather challenging.

Au nanostructures with excellent biocompatibility have been attracted considerable attention in biomedical applications. Such materials can exhibit high antibacterial property against a wide spectrum of bacteria and could be functional with high-density antimicrobial peptides or small molecule antibiotics (Rai et al., 2016; Yang et al., 2017). In addition, the size-dependent effects of biological activity indicate novel physicochemical properties at the nanoclusters (NCs) scale. Zhang’ group (Zhang et al., 2019) reported the excellent antibacterial efficiency of AuNCs against *S. aureus*, *S. epidermidis*, and *P. aeruginosa* by producing intracellular ROS, and the antibacterial activity could be controllably regulated via the surface properties. Zheng’s group synthesized the 6-mercaptohexanoic acid (MHA)-modified sub-2 nm AuNCs with high antibacterial against both Gram-positive and Gram-negative bacteria, which showed a great advantage over that of large-size gold NPs (Zheng et al., 2017). As the surface properties of AuNCs play the major role in their antibacterial activity, AuNCs functionalized with a series of surface ligands have been investigated to obtain high antibacterial activity. Thus, 4, 6-diamino-2-mercaptopyrimidine (DAMP), an analog of 2-mercaptopyrimidine in the tRNA of *E. coli*, has been employed as the surface ligand to functionalize AuNCs. The DAMP-capped AuNCs show a robust capacity to eliminate bacteria through DNA destruction, membrane damage, and ROS production (Zheng et al., 2018). Importantly, the AuNCs have great biocompatibility and antibacterial efficiency, indicating that they are suitable for clinical infection therapy. However, AuNCs can be easily aggregated due to their high specific surface area. Various materials including graphene, silica, metal oxide, and polymers, have been used as the substrate materials to load the AuNCs in order to prevent their aggregation (Ji et al., 2013). Presently, only a few studies considered AuNCs as the precursor to construct the antibacterial CNF film (Lin and Alain, 2014; Guo et al., 2016). Hence, we hypothesized that coupling the AuNCs with CNF can vest the antibacterial property to the CNF film and provide a remarkable substrate material for AuNCs.

In this study, the AuNCs were synthesized as antibacterial ingredient for CNF film and their structural features were characterized. Various amount of AuNCs were added into CNF dispersion to obtain the antibacterial CNF films that were termed as AuNCs@CNF films. The effects of different additions of AuNCs on the morphology, crystallinity, mechanical, thermal, and surface wettability were investigated. The *in vitro* and *in vivo* antibacterial properties of AuNCs@CNF films were evaluated by prohibiting the growth of bacteria (*E. coli* and *S. mutans*) and the skin regeneration of rats by infecting with the bacteria, respectively. Thus, we speculated that the AuNCs@CNF film with great antibacterial activity both *in vitro* and *in vivo* could promote the biomedical application of CNF film.

## Materials and Methods

### Materials and Reagents

HAuCl_4_⋅3H_2_O and mercaptopyrimidine were purchased from Sigma-Aldrich Co., Ltd. Ultrapure water was obtained from Millipore Autopure system. The bleached hardwood kraft pulp that used as the raw material to prepare the CNF film was provided by Asia Symbol Pulp and Paper Co., Ltd (Shandong, China). The used 2, 2, 6, 6-tetramethylpiperidine-1-oxyl radical (TEMPO), sodium bromide (NaBr), and sodium hypochlorite (NaClO) were analytical grade without purification.

### Synthesis and Characterization of AuNCs

The mercaptopyrimidine-modified AuNCs were prepared as described previously (Zheng et al., 2018). Briefly, 254 mg of mercaptopyrimidine was solubilized in 2 mL of 50% ethanol solution. It was mixed with 1 mL of 10 mM chloroauric acid trihydrate solution in 7 mL ultrapure water and heated to 70°C, followed by continuous stirring at 300 rpm for 12 h before cooling to room temperature. The synthesized AuNCs were purified by centrifuging at 10000 rpm for 10 min to remove bulk gold and dialyzed in membrane tubing (MWCO = 5000) against ultrapure water to remove the excess mercaptopyrimidine molecules. Finally, the as-prepared AuNCs were stored at 4°C until further use. The morphology of AuNCs was characterized by transmission electron microscopy (TEM, JEM-2100, Japan) and field-emission scanning electron microscope (FE-SEM, Zeiss Supra 40 Gemini, Germany). The hydrodynamic size of AuNCs was measured by dynamic light scattering (Malvern Zetasizer Nano ZS90, United Kingdom).

### Preparation of CNF Dispersion, CNF Film, and AuNCs@CNF Films

The bleached kraft pulp was treated by the NaClO/NaBr/TEMPO system to obtain the TEMPO-oxidized CNF, as described in the work of Yu et al. (2019). The oxidized CNF solution was diluted by distilled water to 0.5% (w/w) concentration and homogenized by the pressured homogenizer at room temperature and 500 bar for 3 cycles to obtain the CNF dispersion. To active the carboxyl group in the TEMPO-oxidized CNF, 0.4 g of EDC and 0.2 g of NHS were dissolved in 200 mL of CNF dispersion and stirred at room temperature for 6 h. The residual EDC and NHS in the CNF dispersion were removed by dialysis (molecular weight cutoff of the membrane was 10000 g/mol). AuNCs solution with the final concentrations of 0.05, 0.1, and 0.2 mM in the CNF dispersion were mixed with active CNF dispersion (0.5%, w/w), respectively, and stirred overnight to obtain the uniform AuNCs@CNF dispersion. The CNF and AuNCs@CNF dispersion were cast into polystyrene Petri dish and dried at 30°C until the films were formed.

### Characterization of the Prepared Films

The morphologies of the prepared films were analyzed by atomic force microscopy (AFM, Signal Hill, CA, United States) and SEM (Carl Zeiss NTS, Hitachi Ltd, Tokyo, Japan) at accelerating voltages of 200 and 15 kV, respectively. The spectra of molecular vibrations of the prepared films were obtained by an ATR-FTIR spectrophotometer (Nicolet iS10, Thermo, Waltham, MA, United States). All the spectra were recorded from 400 to 4000 cm^–1^ with 32 scans at a resolution of 4 cm^–1^. X-ray diffraction (XRD) was performed to evaluate the crystallinity (CrI) properties of the prepared films. The spectra were recorded from 5 to 50° with 4°/min scanning rate at a voltage of 40 kV and a current of 40 mA. The Crl of the prepared films was calculated according to the equation described by Sunghyun work (Sunghyun et al., 2016). The light transmittance spectra of the films were recorded in the range of 300–800 nm using a UV-vis spectrophotometer (Ultraspec 2100, Amersham Bioscience). The transmittance percentages of the films were calculated according to Beer–Lambert Law at the normalized wavelength (400 nm) for the film with similar thickness. The thermogravimetric analysis (TGA) of the prepared films was carried out by a thermogravimetric analyzer (PerkinElmer, Inc., Waltham, MA, United States). The film samples were heated from 30 to 600°C with a heating rate of 10°C/min under nitrogen atmosphere with a gas flow of 20 mL/min. The mechanical properties (tensile strength, tensile strain, and Young’s modulus) of the prepared films were measured using a universal material testing machine (Shimadzu Co., Japan). The films were cut into rectangular specimens (length 50 mm, width 5 mm, thickness 0.02–0.04 mm) for tensile testing. The span length and testing speed were 30 mm and 1 mm/min, respectively.

### Antibacterial Activity Analysis

The antibacterial activity of the CNF and AuNCs@CNF dispersion were tested using broth dilution method. *Escherichia coli* (*E. coli*, ATCC-25922) and *Streptococcus mutans* (*S. mutans*, ATCC-21059) were cultured at a density of 1.0 × 10^6^ CFU/mL in 1 mL dispersions. The as-prepared bacterial solutions were inoculated at 37°C for 24 h. The reduction in the number of bacteria in the medium were calculated to evaluate the antibacterial activity. The antibacterial activity of the CNF and AuNCs@CNF films were tested using the inhibition zone method. A volume of 200 μL (10^6^ spores/mL) of *E. coli* and *S. mutans* were homogeneously swabbed on the surface of the agar media. Then, the prepared films were cut into circles of 0.6 cm in diameter and placed on the agarose gel and incubated at 37°C for 24 h. The images of the plates were recorded to measure the inhibition zone for the bacteria.

The morphological properties of the bacteria treated with CNF and AuNCs@CNF dispersion were observed by FE-SEM. Specifically, the bacterial solutions cultured with the dispersions were harvested by centrifugation at 8000 rpm for 5 min and washed three times with PBS after culturing with AuNCs dispersion, followed by the addition of 2.5% glutaraldehyde at 4°C for 12 h to allow complete immobilization. Subsequently, the bacteria were dehydrated with graded ethanol and resuspended by absolute ethyl alcohol. Finally, the immobilized bacterial solutions were dropped on a silicon wafer (10 mm × 10 mm) and dried in a vacuum drier for FE-SEM analysis.

### Animal Experiment

Twenty-four Male Sprague–Dawley rats (weight ∼250 g) were obtained from the Laboratory Animal Center of Drum Tower Hospital Affiliated to the Medical School of Nanjing University (China). All experimental protocols in this study were approved by the Committee of Drum Tower Hospital Affiliated to Medical School of Nanjing University. To establish SD rat skin infection model, two skin defects (10 mm × 10 mm) were made on the back of the animals, followed by the inoculation of 200 μL *E. coli* or *S. mutans* (1 × 10^9^ CFU/mL). Then, the neat CNF and AuNCs@CNF size-matched films were attached to the corresponding skin defects in the experiment and the blank control groups (without any control). After 4 or 8 days post-treatment, the area of the wounds and the histological sections were observed for the *in vivo* evaluation of the antibacterial activities of the CNF and Au NCs@CNF films.

### Statistical Analysis

All experiments for the antibacterial analysis were performed with three replicates. All the results were expressed as mean ± standard deviation. Statistical analysis was performed using Origin software (8.5 version). Asterisks in the statistical analysis indicate the statistically significant differences between control and experience groups (^∗^*p* < 0.05; ^∗∗^*p* < 0.01; ^∗∗∗^*p* < 0.005;^****^p < 0.001).

## Results

### Synthesis and Characterization of AuNCs

**FIGURE 1:**
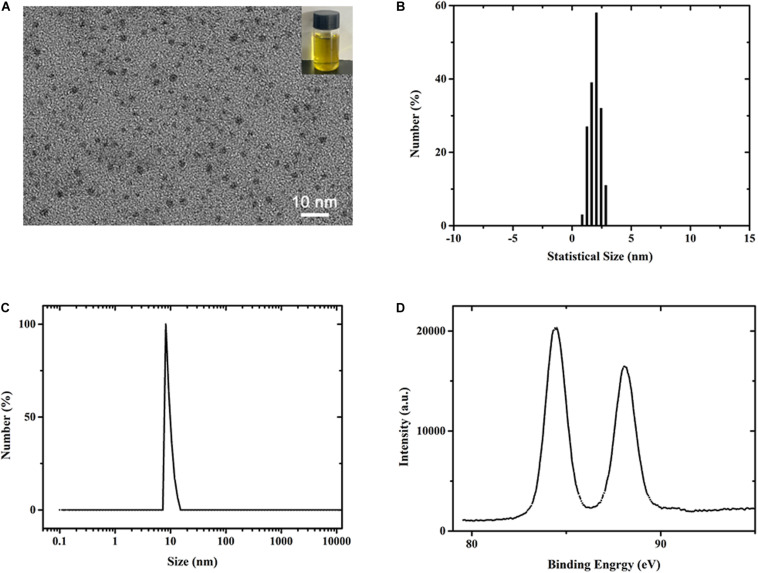
**(A–C)** TEM image of DAMP modified AuNCs. The inset presents the photograph of the Au NCs solution **(B)** Statistical size distribution of AuNCs in **(A)**. **(C,D)** DLS and XPS spectra measurement of synthesized AuNCs.

The mercaptopyrimidine-modified AuNCs were synthesized by reducing chloroauric acid in the presence of mercaptopyrimidine ligands in 50% ethanol solution. The morphology of the prepared AuNCs was characterized by TEM as shown in Figure 1A. Photograph of as-prepared AuNCs solution was shown in Figure 1A (inset), which showed fluorescent yellow under visible light. The as-synthesized AuNCs were ultrasmall spherical clusters showing a good monodispersity. According to the TEM image, it can be found that the average size of AuNCs was 1.93 ± 0.21 nm (Figure 1B). These results suggested that the obtained AuNCs was present in nanostructure state (Zheng et al., 2019). The data from dynamic light scattering (Figure 1C) demonstrated that the average hydrodynamic size of AuNCs was about 10 nm with a polydispersity index of 0.153, indicating their great dispersibility. Also, the AuNCs solution was highly stable and could be stored at 4°C without visible precipitation and flocculation. To further investigate the valence states of Au in AuNCs, X-ray photoelectron spectroscopy (XPS) measurement was performed for the AuNCs aqueous solution. The spectrum (Figure 1D) showed that the binding energy (EB) for Au (4f_5/2_) and Au (4f_7/2_) were 88.1 and 84.4 eV, respectively, indicating that the Au occurred in both zerovalent and monovalent state. In addition, biocompatibility of the as-prepared AuNCs was tested in human bone marrow-derived mesenchymal stem cells (hBMSCs) and rat bone marrow mesenchymal stem cells (rBMSCs). After treated with AuNCs for 72 h, almost no obvious morphology changes were observed in rBMSCs and hBMSCs (Supplementary Figure S1). Cell Counting Kit-8 (CCK-8) was performed for testing the potential cytotoxicity of cells after treatment with AuNCs for 1, 3, and 5 days. The results for rBMSCs and hBMSCs (Supplementary Figure S2) unarguably confirmed that AuNCs showed almost no cytotoxicity, with a good cell viability over 95% even at a concentration up to 0.2 mM. Overall, the aforementioned results indicated the as-prepared DAMP-modified AuNCs were the expectant substance that can be used as the ingredient to fabricate the antibacterial nanocomposite films.

### Antibacterial Activity of As-Prepared AuNCs@CNF Dispersion

**FIGURE 2:**
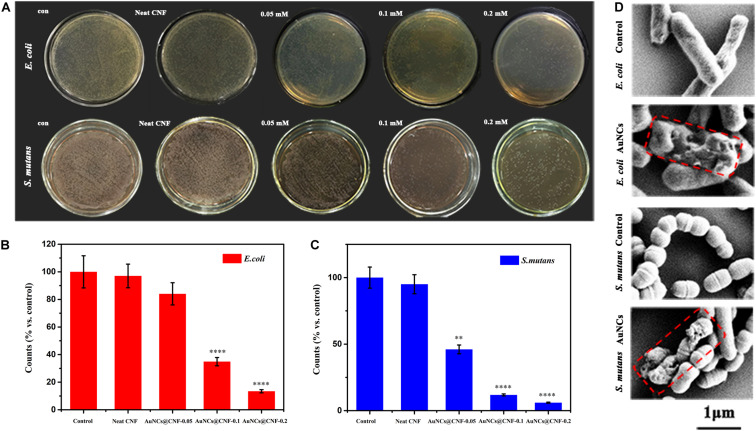
**(A)** Photographs and **(B,C)** corresponding statistics of bacterial colonies (*E. coli* or *S. mutans*) formed on LB-agar plates after cultivating with AuNCs@CNF dispersion solutions. **(D)** SEM micrographs of *E. coli and S. mutans* when treated or untreated with AuNCs. Asterisk indicates statistically significant differences between control and experience group (^∗∗^*p* < 0.01; ^****^p < 0.001).

The present study aimed to add the AuNCs in the CNF film to enhance its antibacterial activity. The effects of the AuNCs@CNF dispersion solutions on the growth of bacteria were shown in Figure 2A. Compared with the positive control group, almost no decrease of bacterial colonies formation was observed in the group with neat CNF dispersion, indicating that neat CNF almost had no antibacterial property. On the contrary, it can be seen that a number of bacterial colonies formed on LB-agar plates decreased when they were treated by AuNCs@CNF dispersion. Specifically, the accrued bacterial counts (Figures 2B,C) demonstrated that *E. coli* or *S. mutans* treated with AuNCs@CNF dispersion solutions had a dose-dependent increase in antibacterial efficiency with the respective decrement degree of 15.96 ± 1.53% or 53.92 ± 3.85% at 0.05 mM AuNCs, 65.12 ± 5.61% or 88.17 ± 5.67% at 0.1 mM AuNCs, 86.58 ± 7.4% or 94.0 ± 5.94% at 0.2 mM AuNCs, as compared to the control group. In order to further investigate the antibacterial mechanism of AuNCs, the morphological properties of the bacteria treated by AuNCs were observed by FE-SEM (Figure 2D). The morphology of the bacterial cells appeared intact and smooth in the control group (without treatment by AuNCs). When treated by 0.2 mM AuNCs at 37°C for 12 h, the bacterial cells showed obvious damage with the wizened membranes.

### Morphological Analysis of the Films

**FIGURE 3:**
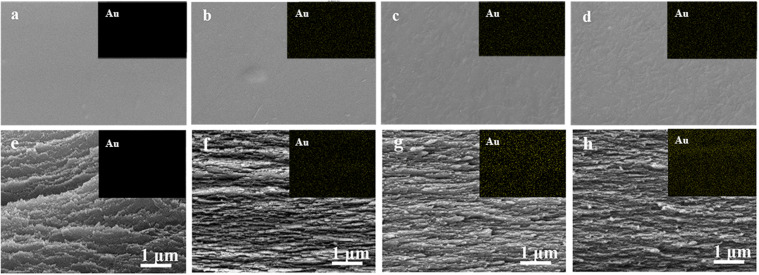
SEM images of surface morphologies of the prepared CNF **(a)** and AuNCs@CNF films with 0.05 mM AuNCs **(b)**, 0.1 mM AuNCs **(c)**, 0.02 mM AuNCs **(d)**, respectively; Cross-sections of prepared CNF **(e)** and AuNCs@CNF films with 0.05 mM AuNCs **(f)**, 0.1 mM AuNCs **(g)**, 0.02 mM AuNCs **(h)**, respectively.

Photographs of as-prepared neat CNF film, AuNCs@CNF-0.05, AuNCs@CNF-0.1, and AuNCs@CNF-0.2 were shown in Supplementary Figure S3, where it can be seen that the neat CNF film was colorless and transparent and became a little primrose yellow after coupling with Au NCs. The surface morphologies were elucidated by SEM (Figure 3). The cellulose nanofibrils (Figure 3a) were homogeneously dispersed in the CNF films without the appearance of the individual fibrils on the surface of the film. For AuNCs@CNF films contained 0.05 mM (Figure 3b), 0.1 mM (Figure 3c), and 0.2 mM (Figure 3d) AuNCs, the introduced nano clusters were homogeneously distributed in the films, as indicated by the SEM-mapping images of the Au element (top right pictures). In addition, the cross-section morphologies of the prepared films were also investigated by SEM. The layered structure of the nanofibrils could be observed in the neat CNF film (Figure 3e) and AuNCs@CNF films (Figures 3f–h). As proved by the cross-section SEM-mapping images of the Au element distributions (top right pictures), the introduced AuNCs were homogeneously distributed in the matrix of cross-section films.

**FIGURE 4:**
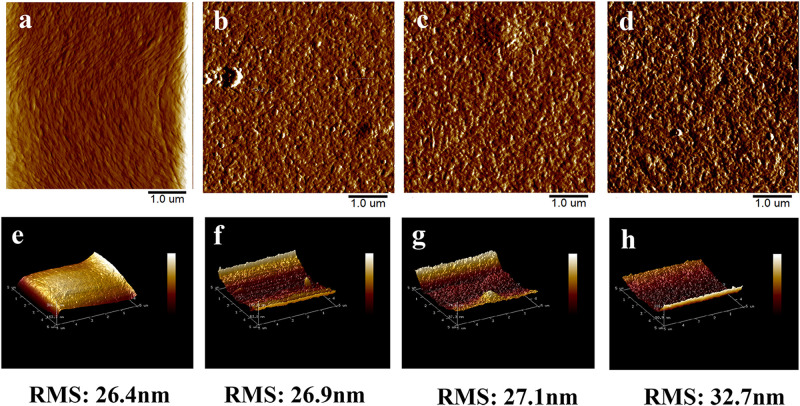
The AFM surface images and 3D images of CNF film **(a,e)** and AuNCs@CNF films with 0.05 mM AuNCs **(b,f)**, 0.1 mM AuNCs **(c,g)**, 0.02 mM AuNCs **(d,h)**.

In order to understand the effect of AuNCs’ addition on the surface properties of the CNF film, the plane view and 3D images of the surface structure of the prepared films were evaluated by AFM (Figures 4a–h). Specifically, the roughness (RMS) value was 26.4 nm for neat CNF film (Figure 4e), while the values for AuNCs@CNF films with increased concentration of AuNCs 0.05–0.2 (Figures 4f–h) increased from 26.9 to 32.7 nm. The increased roughness of the prepared AuNCs@CNF films might be due to the degree of consolidation on the surface during drying process for the film forming (Rojo et al., 2015), which was in agreement with the observation of the SEM images (Figure 3).

### Basic Characterization of CNF and AuNCs@CNF Films

**FIGURE 5:**
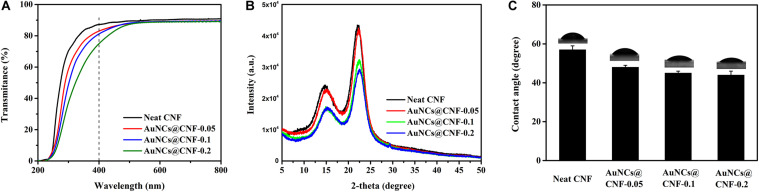
The light transmittance **(A)**, XRD patterns **(B)** and water contact angles **(C)** of the prepared films.

Transparency is the optical property for the CNF film (Mehta and Kumar, 2019; Yu et al., 2019). Hence, the light transmittances of prepared CNF and AuNCs@CNF films were measured (Figure 5A). The transparency in visible light spectra of AuNCs@CNF films showed a decline with the increased AuNCs concentration. In order to quantitatively understand the effect of AuNCs on the visible light transmittance of CNF, the light absorption of the normalized films with similar thickness (10 nm) was calculated according to the Beer–Lambert Law at 400 nm (Table 1). It can be seen that the transmittance of normalized light of AuNCs@CNF-0.05, AuNCs@CNF-0.1, and AuNCs@CNF-0.2 films were 69.6, 70.1, and 63.4%, respectively, which was lower than that of neat CNF film (73.8%).

**TABLE 1:** Properties of transmittance, crystallinity, and thermal stability of the prepared films.

Film abbreviation	Transmittance (%, at 400 nm)	Crystallinity (%)	Thermal property		
			Tonset (°C)	Tmax (°C)	Residual weights (%)
Neat CNF	73.8	63.1	187	242	23.7
AuNCs@CNF-0.05	69.6	63.0	187	254	25.8
AuNCs@CNF-0.1	70.1	62.4	199	256	26.8
AuNCs@CNF-0.2	63.4	59.3	188	261	31.7

To further understand the effects of AuNCs on structural changes of CNF film, the crystal structures of the prepared films with and without AuNCs were investigated by XRD analysis (Figure 5B). Consequently, two sharp signals were detected at 15 and 22.5°, which are the diffraction planes of typical cellulose I (Jia et al., 2017). The diffraction patterns of AuNCs@CNF films were similar to that of neat CNF film, indicating that the addition of AuNCs did not change the crystal type of cellulose. Table 1 shows that the addition of AuNCs from 0.05 to 0.2 mM reduced the crystalline structure of neat CNF film from 63.1 to 63.0–59.3%.

Water mobility plays a critical role in the antibacterial film when it is used as wound healing (Farooq et al., 2019). Hence, the surface wettability of the prepared films were evaluated by water contact angles (Figure 5C). The contact angle of CNF films was 57°. When the added AuNCs in AuNCs@CNF films increased from 0.05 to 0.2 mM, the contact angle was gradually decreased from 48 to 44°, which was attributed to fact that the added AuNCs have abundant mercapto groups that can improve the hydrophilic property of the resultant films.

### Thermal Property of the Prepared Films

**FIGURE 6:**
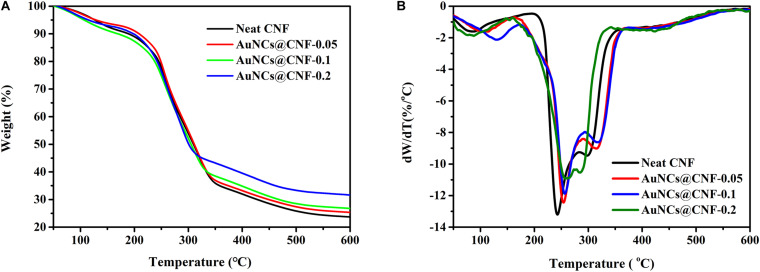
The weight loss curves **(A)** and derivative thermogravimetry curves **(B)** of the prepared films.

Thermal stability of the prepared films is a critical property for considering its applicability in different areas (Bian et al., 2019; Wu et al., 2019b). Hence, the thermal properties of CNF and AuNCs@CNF films were analyzed by TGA. The obtained weight loss curves and derivative thermogravimetry (DTG) curves of the films were shown in Figures 6A,B, respectively. The onset decomposition temperature (Tonset) and maximum decomposition temperature (Tmax) were used as indicators to evaluate the thermal stability of the prepared films (Table 1). According to Figure 6A, the weight loss curves of the films can be divided into three stages. The first stage at 50–150°C was attributed to the evaporation of the absorbed moisture in the films. The second stage at 200–350°C was mainly caused by the decomposition of cellulose in the films. The third stage at 350–600°C was due to the cracks in the thermal degradation intermediates generated in the second stage (Bian et al., 2019; Abral et al., 2020). In this stage, the thermal degradation of the films were relatively stable, and the residual weights were generated. Table 1 also demonstrated the residual weights of CNF film, AuNCs@CNF-0.05, AuNCs@CNF-0.1, and AuNCs@CNF-0.2 films were 23.7, 25.8, 26.8, and 31.7% at 600°C, respectively. Among the four samples, the CNF film displayed the lowest onset temperature (187°C) and the lowest weight loss temperature (242°C). For the AuNCs@CNF film, the increased concentration from 0.05 to 0.2 mM resulted in the prepared film with higher values of Tonset and Tmax, which were 188–199°C and 254–261°C, respectively.

### Mechanical Properties of the Prepared Films

The mechanical properties of cellulose film are the crucial index for its applications (Rojo et al., 2015; Yu et al., 2019; Pei et al., 2020). Hence, the stress-strain curves of the prepared films were evaluated and shown in Supplementary Figure S4. The tensile strength, breaking strain, and elastic modulus of the films were calculated from the curves and shown in Table 2. The neat CNF film had a tensile strength of 108.5 MPa at break, a tensile strain of 5.0%, and Young’s modulus of 5.9 GPa. While, the mechanical properties of CNF film were remarkably influenced by the addition of AuNCs. All AuNCs@CNF films showed the decreased tensile strength and tensile strain of 43.2–54.1 MPa and 4.1–4.7%, respectively. Typically, the tensile properties of the cellulose film are related to single fibrils and interfibrillar bonding, which are linked to the chemical composition, aspect ratio, and the added ingredient (Wang et al., 2018; Imani et al., 2019). Moreover, the CNF films consisted of AuNCs at the concentration of 0.05–0.1 mM had the lower Young’s modulus (4.7–2.1 GPa) than that of neat CNF film (5.9 GPa).

**TABLE 2:** Mechanical properties of CNF film and AuNCs@CNF films.

Film abbreviation	Tensile strength (MPa)	Elongation at break (%)	Young’s modulus (GPa)
CNF	108.5 ± 11.3	5.0 ± 0.6	5.9 ± 1.7
AuNCs@CNF-0.05	54.3 ± 3.1	4.7 ± 0.1	4.7 ± 0.4
AuNCs@CNF-0.1	47.3 ± 2.8	4.2 ± 0.2	4.1 ± 0.3
AuNCs@CNF-0.2	43.2 ± 8.2	4.1 ± 1.2	2.1 ± 0.8

### *In vitro* and *in vivo* Antibacterial Behavior of AuNCs@CNF Film

**FIGURE 7:**
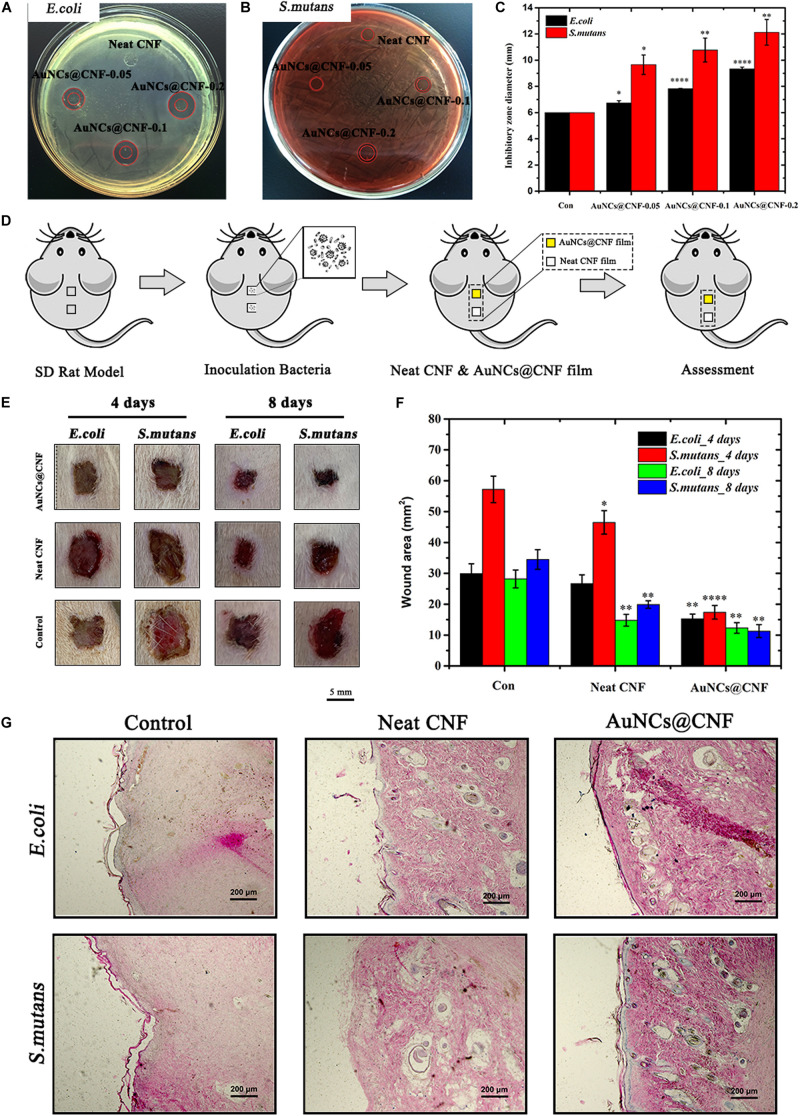
Inhibition zones on the agar plates inoculated with **(A)** *E. coli* and **(B)** *S. mutans* and **(C)** corresponding statistics their diameter. **(D)** Schematic of *in vivo* evaluation process of antibacterial property using skin infected wound model. **(E)** Representative photographs and **(F)** corresponding statistics area of the *E. coli* and *S. mutans* infected wound untreated and treated with AuNCs@CNF films as antibacterial dressings, respectively. **(G)** Histological analysis by H&E staining images of infected wounds covered with neat CNF and AuNCs@CNF films on days 14, respectively. Asterisk indicates statistically significant differences between control and experience group (^∗^*p* < 0.05; ^∗∗^*p* < 0.01; ^****^p < 0.001).

In order to understand the antibacterial effectiveness of as-prepared AuNCs@CNF films, the *in vitro* and *in vivo* assays were performed using bacteria (*E. coli* and *S. mutans*) models and Sprague-Dawley (SD) rats skin infection model, respectively. In Figures 7A,B, the AuNCs@CNF film showed remarkable antibacterial activity for prohibiting the growths of bacteria, as deduced by the formation of inhibitory zones of 6–11.2 mm for *E. coli* and 6–9.2 mm for *S. mutans*. The corresponding statistical data (Figure 7C) demonstrated that AuNCs@CNF-0.2 film displayed relatively larger zones than that of AuNCs@CNF-0.05 and AuNCs@CNF-0.1. To further investigate their antibacterial mechanism, AuNCs release from AuNCs@CNF film were measured by incubating with PBS buffer at 37°C for 24 and 48 h, the results indicated that the AuNCs@CNF had a dose- and time-dependent increase of AuNCs release (Supplementary Figure S5), which play an important role in antibacterial efficiency of AuNCs@CNF films through membrane damage of bacteria (Figure 2D). Surprisingly, the AuNCs@CNF films showed great stability even incubated with PBS buffer up to 48 h as shown in Supplementary Figure S6, which could be used for infected-wound dressing materials.

In addition, *in vivo* antibacterial efficiency of AuNCs@CNF film was investigated via the SD rat skin infection model, in which the healing degree of the bacteria-tainted wound was evaluated by pasting the film on it. In order to facilitate the assessment, SD rats with two dorsal skin defects (10 mm × 10 mm) were randomly divided into three groups (*E. coli* infected group, *S. mutans* infected group, and the blank control group), wherein the upper dorsal skin defect was employed for the antibacterial evaluation of the AuNCs@CNF film and the lower for neat CNF film (Figure 7D). The treatment effect was evaluated by measuring the area of the skin wound every 4 days and the visual images are shown in Figure 7E. The healing process of the wound in the AuNCs@CNF film group was faster than that of the neat CNF film and control group. The corresponding statistics (Figure 7F) showed that the area of wounds in the AuNCs@CNF film group after 8 days of treatment was 12.3 ± 1.7 and 11.3 ± 2.1 mm^2^ for *E. coli* and *S. mutans*, respectively, which were significantly smaller than that of the control group (28.2 ± 2.9 and 34.5 ± 3.2 mm^2^, respectively). In order to further validate the effects of AuNCs@CNF films on wound healing, histological examinations were performed by H&E staining on regenerated skin tissue in the wounds treated with neat CNF films and AuNCs@CNF films 14 days post operation, respectively. As shown in Figure 7G, a well-developed vascular network and new hair follicle were observed in AuNCs@CNF film treated group, whereas it was absent in control group.

## Discussion

CNF-based materials with great antibacterial properties are considered to be very promising platforms for biomedical applications such as food packing films, infected wound-healing formulation and multifunctional antibacterial films (Li et al., 2018; Han et al., 2019). There are several emerging approaches have been carried out to endow CNF-based materials with antibacterial property (Yuan et al., 2018; Chen et al., 2019). In this work, the biocompatible AuNCs were synthesized and used as intermediate to prepare the AuNCs@CNF dispersion for manufacturing film. The prepared dispersions with different concentration showed the antibacterial ability to inhibit the growth of infectious bacteria of *E. coli* and *S. mutans*. Through the bacterial morphologies observed from SEM images, it can be seen that AuNCs could interact with membranes of bacterial cell and destroy its integrity, which might be one of the attributions for the antibacterial property of AuNCs. Based on these results, it is speculated that the dispersions containing AuNCs are promising to prepare the antibacterial films.

Morphological analysis by SEM indicated that the introduced AuNCs with groups of NH_3_ and COOH can form hydrogen bonds with OH group among nanofibers in CNF, resulting in a slick surface for the obtained AuNCs@CNF films. Moreover, the increased amount of AuNCs made the surface of the prepared films rather uneven, which could be attributed to the presence of the surface-active functional groups in the AuNCs particles that can be linked to nanofibrils in the CNF composites (Bian et al., 2019). This can be explained that the AuNCs can cause the flocculation of nanofibrils and disrupt the interfibrillar hydrogen bonding during the dried process for AuNCs@CNF film forming, leading to the uneven surface of the prepared films (Imani et al., 2019). In addition, AuNCs can facilitate to form a relatively compact structure of AuNCs@CNF films, which can be indicated by more compact and flat structures appeared in the AuNCs@CNF films with the increased amount of introduced AuNCs particles. In addition, the AFM surface images of CNF film showed that the neat CNF film contained the nanometer scale cellulose fibrils, which were interconnected to form the fibrous network. This result was in accordance with the work in Yu et al. (2019). When the AuNCs were introduced to the CNF film, the cellulose fibrils in the AuNCs@CNF films were still presented as the fibrous network structure. Overall, the morphology analysis indicated that the addition of AuNCs did not significantly affect the nanostructural features of CNF film.

We further evaluated the basic properties of AuNCs@CNF films, such as transparency, crystal structure, water mobility, thermal stability and mechanical properties. The decreased light transmittance of AuNCs@CNF film was attributed to the surface mercapto group of AuNCs, which is a chromophore that can absorb light and reduce the transmittance of neat CNF film. In addition, the decreased transparency of the AuNCs@CNF film can also be attributed to its enhanced surface roughness (Nogi et al., 2009). Based on the Crl values of prepared films, it can be speculated that the addition of AuNC increased the proportion of amorphous regions of nanofibers in the CNF films (Bian et al., 2019). Even the added AuNCs in CNF films could slightly decrease its contact angle from 57 to 48–44°, the prepared AuNCs@CNF films could be still used as the antibacterial film in the wound field. As pointed out by Kaygusuz et al. (2017), the moderately wettable surface of film with contact angle of 35–50° is benefit for bacteria to adhere, which can improve the contact opportunity between bacteria and antibacterial film. Yang et al. (2016) showed that the enhanced hydrophilic properties of modified cellulose could make contribution to its antimicrobial activity.

From the results of mechanical property analysis, it is found that the added AuNCs in CNF film could slightly reduce its tensile strength, which can be attributed to the weakened interactions between interfibrils in CNF films. Meanwhile, the decreased Crl of the AuNCs@CNF film is another reason for the reduced tensile strength of the CNF film (Bian et al., 2019). In a reported work, the wound dressings from chitosan-cellulose nanocrystals with tensile strength of 34.9–58.0 MPa (Naseri et al., 2015), bacterial cellulose-chitosan membranes with tensile strength of 10.26 MPa (Lin et al., 2013) and 22.48 MPa (Savitskaya et al., 2017), and cellulose-polymer-Ag nanocomposite with 25.1–39.8 (Raghavendra et al., 2013) were successfully prepared and used as antibacterial wound dressing. Hence, it can be speculated that the prepared AuNCs@CNF films with tensile strength of 43.2–54.1 MPa can also perform the comparative mechanical properties than the other wound dressing materials. According to the Young’s modulus results, the flexibility of the CNF film could be enhanced by the addition of AuNCs. Generally, the excellent mechanical properties of high strength and flexibility of the films are required for the most advanced material design. However, the enhancement of strength and flexibility for the cellulose films are usually conflicted (Wang et al., 2018). The Young’s modulus can be regarded as the indicator to evaluate the stiffness and resistance of a material for deforming (Azevedo et al., 2013). The Young’s modulus of wound healing with high value might make it against for skin applications. Comparing to a reported work, it can be found that the Young’s modulus of AuNCs@CNF films had the comparative values than that of wound healing from various bacterial cellulose-based composites, which had the Young’s modulus of 4.2–6.1 GPa (Ul-Islam et al., 2012) and 7.0 GPa (Kim et al., 2011). In the present study, although the tensile strength of the AuNCs@CNF film was reduced with the increasing addition of AuNCs, the enhanced flexibility indicated that the AuNCs@CNF film possessed high folding strength, making it resistant to fracture (deformability) when used as the antibacterial film for application as the dressing over wound surface (Li et al., 2018; Wang et al., 2018).

*In vitro* and *in vivo* antibacterial activity of AuNCs@CNF films were further investigated. The reduced inhibitory zones indicated that the added AuNCs in the CNF film ascribed it possessing significantly inhibiting ability for the growth of both *E. coli* and *S. mutans*. These results were in accordance with the antibacterial activity of their dispersion solutions. In the work of Raghavendra et al., 2013, they found that the cellulose-Ag nanocomposite showed antimicrobial efficacy with inhibition zone of 1.7 mm for *E. coli*. Di et al. (2017) prepared bacterial cellulose/Ag and found it could produce a clear inhibition zones of 25 mm for *E. coli*. The comparative values of inhibitory zones achieved from AuNCs@CNF films indicated that they had the considerably antibacterial behavior as Ag-based cellulose films. The wound healing results revealed that the prepared AuNCs@CNF films showed the abilities to facilitate the healing degree of the bacteria-tainted wound (*in vivo*), which might be due to its antibacterial activity. Generally, the wound healing can be impede by various factors, such as the population of microorganisms, existence of multidrug-resistant microorganisms and formation of bacterial microcolonies (Simoes et al., 2018). Hence, it can be speculated that the prepared AuNCs@CNF film with high antibacterial property has great potential in traumatic infection therapy and promoting bacteria-tainted skin healing.

## Summary

In this study, the synthesized biocompatible Au nanoclusters showed great antibacterial properties for the prohibition of *E. coli* and *S. mutans* when it was in CNF dispersion solution. When the AuNCs were coupled with CNF film, the obtained the AuNCs@CNF films had improved hydrophilicity, flexibility, and thermal stability than that of neat CNF film. The prepared AuNCs@CNF films could prohibit the growth of bacteria (*E. coli* and *S. mutans*) *in vitro*. In addition, the AuNCs@CNF film showed the ability to heal the bacteria-tainted wound of rat skin *in vivo*. The AuNCs@CNF film with great antibacterial activity indicated it possessed the potential application in biomedical field.

## Data Availability Statement

The raw data supporting the conclusions of this article will be made available by the authors, without undue reservation.

## Ethics Statement

The animal study was reviewed and approved by the Ethics Committee of Drum Tower Hospital affiliated to the Medical School of Nanjing University.

## Author Contributions

CH and QJ proposed the idea. PW and BY did the experiments. HD helped the property evaluation of CNF film. YiZ helped the skin repair experiment. YaZ, RC, and ZY helped the antibacterial evaluation of AuNCs and AuNCs@CNF films. All authors contributed to the article and approved the submitted version.

## Conflict of Interest

The authors declare that the research was conducted in the absence of any commercial or financial relationships that could be construed as a potential conflict of interest.
