# Supplementary material for: Coupling Biocompatible Au Nanoclusters and Cellulose Nanofibrils to Prepare the Antibacterial Nanocomposite Films
Source: Front Bioeng Biotechnol. 2020 Aug 18;8:986. doi: 10.3389/fbioe.2020.00986 (PMC7466770; doi:10.3389/fbioe.2020.00986)
Supplement: Supplementary file 1 [file Image_1.pdf]

Supplementary Material

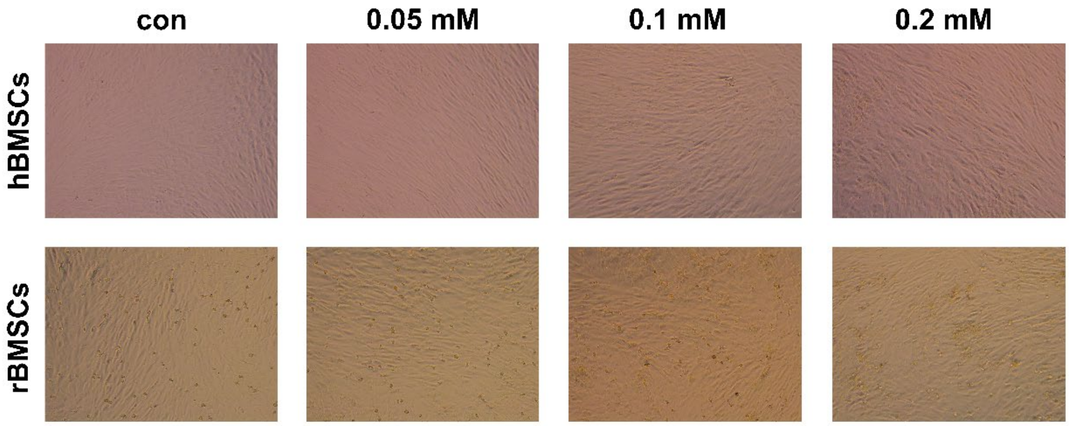

Supplementary Figure S1. Photographs of hBMSCs and rBMSCs treated with AuNCs with concentration of 0.05 mM, 0.1 mM and 0.2 mM respectively.

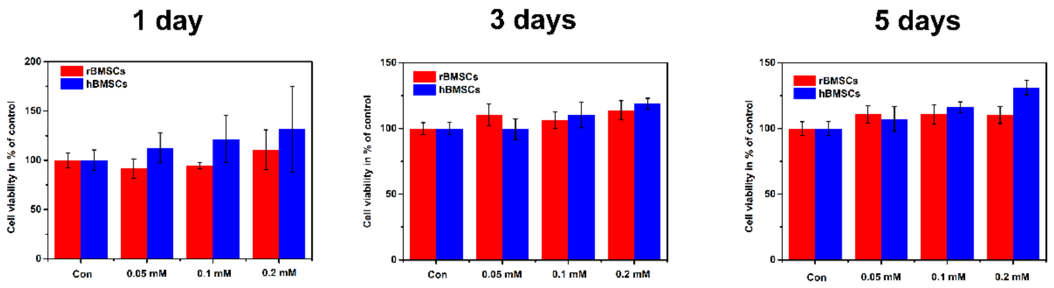

Supplementary Figure S2. Cell viability of rBMSCs and hBMSCs cells after incubated with AuNCs for 1, 3 and 5 days respectively.

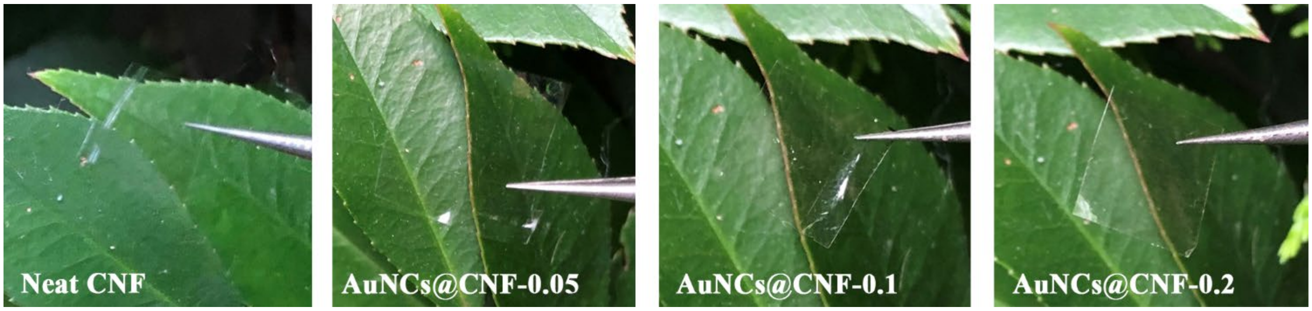

Supplementary Figure S3. Photograph of as-prepared neat CNF film, AuNCs@CNF-0.05, AuNCs@CNF-0.1 and AuNCs@CNF-0.2 respectively.

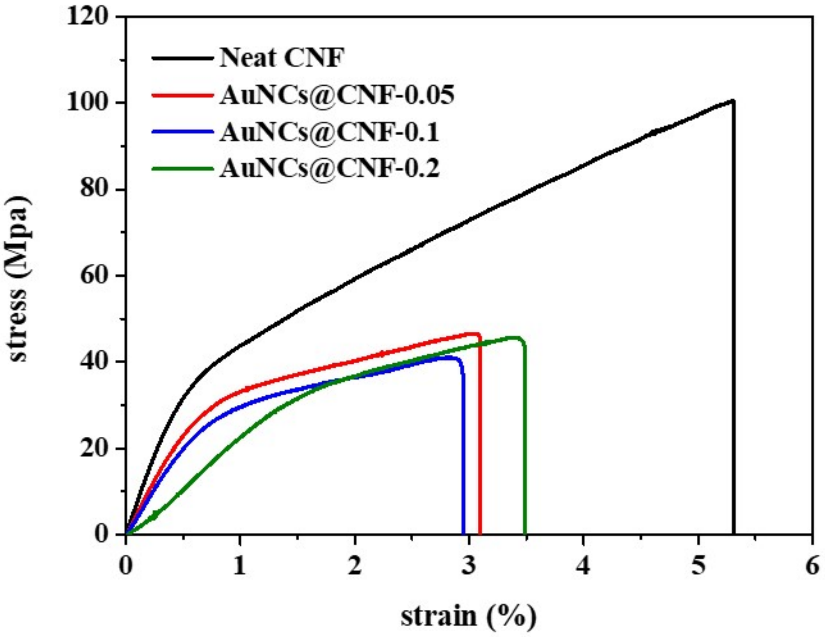

Supplementary Figure S4. Stress-strain curves of the prepared films

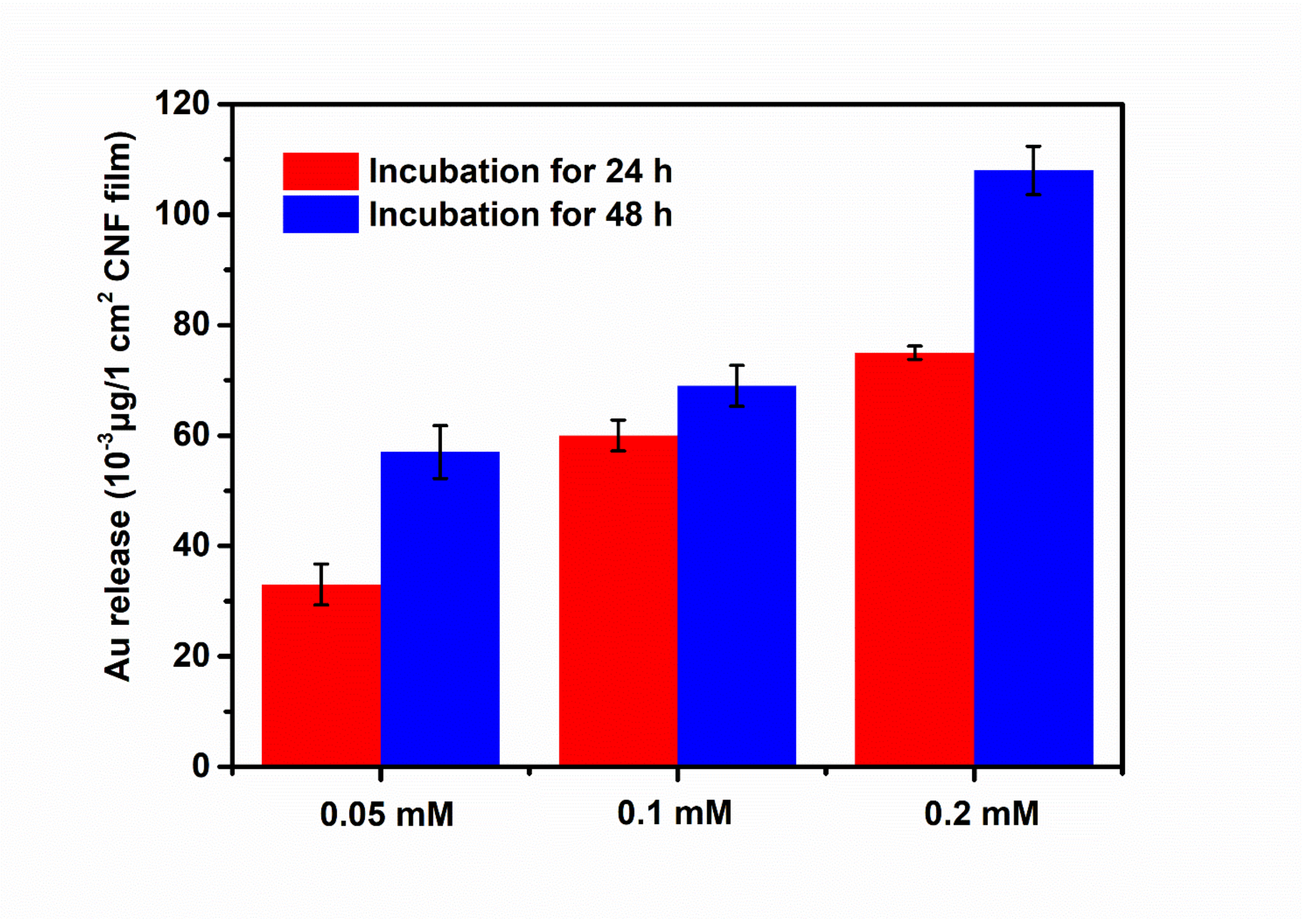

Supplementary Figure S5. AuNCs release from AuNCs@CNF films by incubating with PBS buffer at 37°C for 24 and 48 h

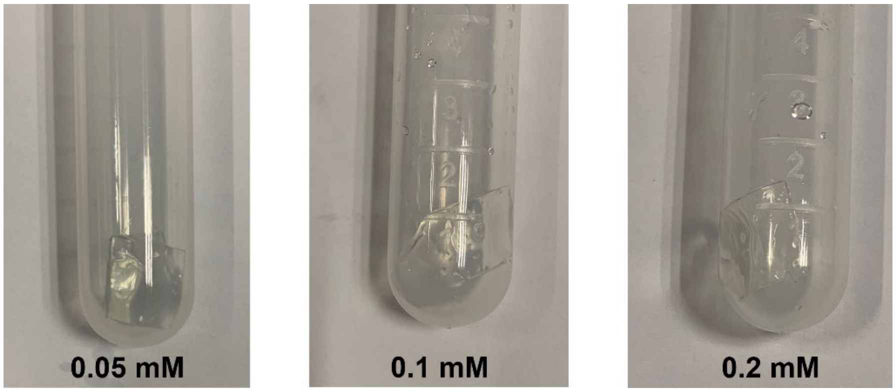

Supplementary Figure S6. Photographs of AuNCs@CNF-0.05, AuNCs@CNF-0.1 and AuNCs@CNF-0.2 films after incubated with PBS buffer at 37°C for 48 h respectively.
